# Association between Coexisting Dementia and Mortality in Patients Receiving Oral Epidermal Growth Factor Receptor Tyrosine Kinase Inhibitors: A Nationwide Real-world Database Study

**DOI:** 10.31662/jmaj.2026-0132

**Published:** 2026-07-31

**Authors:** Masaki Sogame, Yasutaka Ihara, Shoichiro Yamamoto, Maya Shimasaki, Tomohisa Kurata, Shinji Akiyama

**Affiliations:** 1 Department of Pharmacy, Ehime Prefectural Central Hospital, Matsuyama, Japan; 2 Clinical Research Promotion Unit, Clinical Therapeutic Trial Center, Ehime University Hospital, Toon, Japan; 3 Center for Data Science, Ehime University, Matsuyama, Japan; 4 Integrated Medical and Agricultural School of Public Health, Ehime University, Toon, Japan; 5 Department of Cardiology, Pulmonology, Hypertension and Nephrology, Graduate School of Medicine, Ehime University, Toon, Japan; 6 Department of Clinical Oncology, Ehime University Graduate School of Medicine, Toon, Japan; 7 Department of Clinical Pharmacy, College of Pharmaceutical Sciences, Matsuyama University, Matsuyama, Japan

**Keywords:** real-world data, dementia, oral epidermal growth factor receptor tyrosine kinase inhibitors

## Abstract

**Introduction:**

Oral epidermal growth factor receptor tyrosine kinase inhibitors (EGFR-TKIs) are standard first-line treatments for EGFR-mutated non–small cell lung cancer (EGFR-m NSCLC). Because these agents require sustained self-management, cognitive impairment may adversely affect treatment continuity and outcomes. However, the association between coexisting dementia and survival in patients receiving EGFR-TKIs remains unclear.

**Methods:**

This cohort study used a large Japanese administrative claims database. Patients aged ≥18 years with newly diagnosed NSCLC who initiated first-line EGFR-TKI therapy between 2018 and 2024 were included. Coexisting dementia was identified using diagnosis codes recorded before treatment initiation. The primary outcome was 3-year all-cause mortality. Cox proportional hazards models were used to estimate adjusted hazard ratios (aHRs) and 95% confidence intervals (CIs), adjusting for age, sex, body mass index, smoking status, comorbidity burden, functional status (Barthel Index [BI]), hypertension, dyslipidemia, and calendar year of treatment initiation.

**Results:**

Among 13,281 eligible patients, 282 (2.1%) had coexisting dementia. During follow-up, coexisting dementia was associated with higher all-cause mortality (aHR 1.35, 95% CI 1.06-1.73). In subgroup-specific analyses, preserved functional status (BI = 100) was associated with lower mortality among patients with dementia, whereas multiple clinical factors were associated with mortality among those without dementia.

**Conclusions:**

Coexisting dementia was associated with poorer survival among patients with EGFR-m NSCLC treated with EGFR-TKIs. Preserved functional status was associated with lower mortality among patients with dementia. These findings suggest that coexisting dementia may identify a clinically vulnerable subgroup in whom functional status warrants careful attention at the initiation of EGFR-TKI treatment.

## Introduction

Epidermal growth factor receptor–mutated (EGFR-m) non–small cell lung cancer (NSCLC) is a distinct molecular subtype for which oral EGFR tyrosine kinase inhibitors (EGFR-TKIs) have become the cornerstone of first-line treatment. Across multiple clinical trials, EGFR-TKIs have yielded substantial improvements in progression-free survival and overall survival compared with cytotoxic chemotherapy, establishing them as standard-of-care therapies worldwide ^(1), (2)^. Therefore, long-term disease control using orally administered targeted agents is becoming increasingly common in routine oncology practice.

However, the real-world effectiveness of EGFR-TKIs is shaped not only by molecular susceptibility but also by patient-level and care process factors that govern treatment delivery and persistence. Unlike intravenously administered chemotherapy, oral targeted therapies shift a substantial proportion of the treatment responsibility from clinicians to patients, requiring sustained medication self-management, adherence to complex dosing schedules, timely management of adverse events, and continuity of outpatient follow-up. Therefore, adherence to oral anticancer agents has emerged as a critical mediator of treatment effectiveness and survival, with nonadherence consistently associated with inferior clinical outcomes ^(3), (4)^.

Dementia can compromise self-management processes. Deficits in prospective memory, executive function, and the instrumental activities of daily living can lead to missed doses, inappropriate treatment interruptions, delayed recognition of adverse events, and fragmented engagement in oncological care ^(5)^. In previous studies, patients with coexisting dementia were less likely to receive guideline-concordant cancer treatment, more likely to experience early treatment discontinuation, and had worse overall survival across multiple cancer types ^(6)^. These disparities may be particularly pronounced for therapies that rely heavily on patient-driven adherence rather than on supervised administration.

Adherence to oral anticancer agents varies widely in routine practice and is strongly influenced by patient-level cognitive, psychological, and social factors ^(3), (7)^. Emerging evidence outside clinical trials suggests that coexisting dementia is associated with lower adherence to EGFR-TKIs, raising concerns that the survival benefits observed in controlled settings may not be fully realized in this vulnerable population ^(8)^. However, whether this cognitive vulnerability translates into diminished survival among patients with EGFR-m NSCLC treated with EGFR-TKIs remains unclear.

To address this knowledge gap, we conducted a large-scale real-world study using administrative claims data to examine the association between coexisting dementia and all-cause mortality in patients with EGFR-m NSCLC receiving EGFR-TKIs. We hypothesized that coexisting dementia would be associated with poorer survival in this population. Furthermore, we performed an exploratory analysis to identify clinical factors associated with mortality among patients with dementia receiving EGFR-TKIs.

## Materials and Methods

### Data source

This study used administrative claims data from advanced treatment centers in Japan. Medical Data Vision Co., Ltd. (MDV; Tokyo, Japan) provided data on patients diagnosed with lung cancer (C34, as per the International Classification of Diseases, 10th Revision [ICD-10]) between April 1, 2008, and December 31, 2024. The MDV database includes more than 50 million patients and represents approximately 28% of hospitals operating under the diagnostic procedure combination/per diem payment system (DPC/PDPS) ^(9)^. This is a case-mix classification–based PDPS that ensures fixed daily compensation based on diagnosis grouping ^(10)^. It has been adopted by most designated acute care hospitals and categorizes patients as having a DPC based on a combination of diagnoses and procedures performed during hospitalization ^(11)^. The dataset included patient information regarding outpatient and inpatient medical care such as basic patient characteristics (age, sex, weight, and height), hospitalization dates, diagnoses based on ICD-10 codes, survival status, and clinical procedures. The MDV database has been used in several studies investigating lung cancer ^(8), (12), (13), (14), (15), (16)^.

### Study population

Patients aged ≥18 years, who received EGFR-TKIs (gefitinib, erlotinib, afatinib, dacomitinib, or osimertinib) for newly diagnosed NSCLC (ICD-10 code C34) between August 2018 and December 2024, were included. The study period was chosen to align with the approval of osimertinib as first-line treatment for EGFR-m NSCLC in Japan. The index date was defined as the date of first EGFR-TKI prescription. To ensure the accurate identification of first-line therapy, patients were required to have at least a 1-month interval between their entry into the database and the date of their lung cancer diagnosis. To restrict the cohort to patients with advanced NSCLC, patients were excluded if they received adjuvant chemotherapy within 60 days before the index date and received chemoradiotherapy within 14 days before the index date.

### Identification of coexisting dementia

Coexisting dementia status was ascertained from the ICD-10 diagnosis codes recorded before the index date. Coexisting dementia was identified using preindex ICD-10 diagnosis codes for dementia syndromes and Alzheimer’s disease (F00, F01, F02, F03, or G30), based on previous claims-based studies ^(17), (18)^. Patients meeting this definition were classified as having coexisting dementia, and all remaining patients were classified as not having dementia.

### Demographic and clinical information

Clinical characteristics were ascertained from data recorded at or before the index date. Based on the information available on the index date, data for age, sex, type of first-line EGFR-TKI, and calendar year of treatment initiation were obtained. Using data recorded within 3 months before the index date, we identified body mass index (BMI), Charlson Comorbidity Index (CCI) ^(19)^, and Barthel Index (BI) ^(20)^. Baseline hypertension and dyslipidemia were identified from the diagnostic codes before the index date. Smoking status (never, current, or former) was determined from the most recently available record before the index date.

### Identification of death

Survival status and date of death were obtained from the discharge summaries. For patients with confirmed death, data up to the date of death were also extracted. For patients with unconfirmed death, follow-up was censored on the date of the last recorded medical procedure. Survival status was tracked for up to 3 years from the index date.

### Assessment of medication adherence and treatment continuation

Medication-taking behavior during EGFR-TKI therapy was evaluated using two measures: medication adherence and treatment continuation. Adherence to EGFR-TKI therapy was assessed using the medication possession ratio (MPR). We evaluated MPR at 1 year and 3 years after treatment initiation. For each time point, the denominator was defined individually as the number of days from treatment initiation to the earlier of the evaluation time point or the censoring date. The numerator was defined as the total number of nonoverlapping days covered by any EGFR-TKI prescription during the corresponding observation period. Switching between EGFR-TKIs was regarded as continuation of treatment, and all EGFR-TKI prescriptions contributed to the calculation of covered days. In addition to analyzing MPR as a continuous measure, adherence was also evaluated dichotomously using an MPR threshold of 0.80.

Treatment continuation was evaluated using time to treatment discontinuation (TTD). TTD was defined as the time from initiation of first-line EGFR-TKI therapy to the earliest of the following events: (1) initiation of a subsequent line of therapy, defined as the start of any lung cancer treatment other than an EGFR-TKI; (2) a gap of more than 60 days after the end date of the last EGFR-TKI prescription; or (3) death. Switching between EGFR-TKIs was allowed and was not considered treatment discontinuation. Patients without any of these events were censored at the last date of follow-up.

### Statistical analyses

Baseline demographic and clinical characteristics were summarized separately for patients with and without coexisting dementia. Categorical variables were presented as counts and percentages, and continuous variables were summarized as medians with interquartile ranges (IQRs).

We applied the Kaplan-Meier method to evaluate all-cause mortality according to coexisting dementia status. Cox proportional hazards regression models were used to estimate the association between coexisting dementia and all-cause mortality. To evaluate the independent association between baseline dementia and all-cause mortality, we fitted multivariable Cox proportional hazards models adjusted for prespecified baseline clinical factors, including sex, age, BMI, smoking status, CCI, BI, hypertension, dyslipidemia, and calendar date of treatment initiation. To further explore whether factors associated with mortality differ according to dementia status, we additionally performed subgroup-specific multivariable Cox proportional hazards analyses separately among patients with coexisting dementia and among those without coexisting dementia.

We applied the Kaplan-Meier method to evaluate TTD according to coexisting dementia status. Cox proportional hazards regression models were used to estimate the association between coexisting dementia and TTD. To evaluate the independent association between baseline dementia and treatment discontinuation, we fitted multivariable Cox proportional hazards models adjusted for the same prespecified baseline clinical factors.

These results were expressed as hazard ratios (HRs) with 95% confidence intervals (CIs). All statistical tests were two-sided, and p < 0.05 was considered statistically significant. Missing values for BMI, smoking status, and BI were handled using multiple imputation with predictive mean matching, generating 10 imputed datasets ^(21)^. As sensitivity analyses for all-cause mortality, we repeated the multivariable Cox proportional hazards regression analyses using complete-case data, including only patients with complete information on all covariates, while adjusting for the same covariates as those included in the corresponding primary models. These complete-case analyses were performed for both the overall analysis evaluating the association between coexisting dementia and all-cause mortality and the subgroup analysis among patients with coexisting dementia. Statistical analyses were conducted using R version 4.3.3 (R Foundation for Statistical Computing, Vienna, Austria) ^(22)^.

## Results

### Study populations and their baseline characteristics

A flow diagram illustrating the patient selection process is presented in Figure 1. Data from 14,862 patients with NSCLC who received EGFR-TKIs as first-line treatment between August 2018 and December 2024, were included. After applying the eligibility criteria, 13,281 patients with advanced NSCLC initiated on first-line EGFR-TKI therapy were included in the final study. Among them, 282 (2.1%) had coexisting dementia at baseline, whereas 12,999 (97.9%) did not.

**Figure 1.**
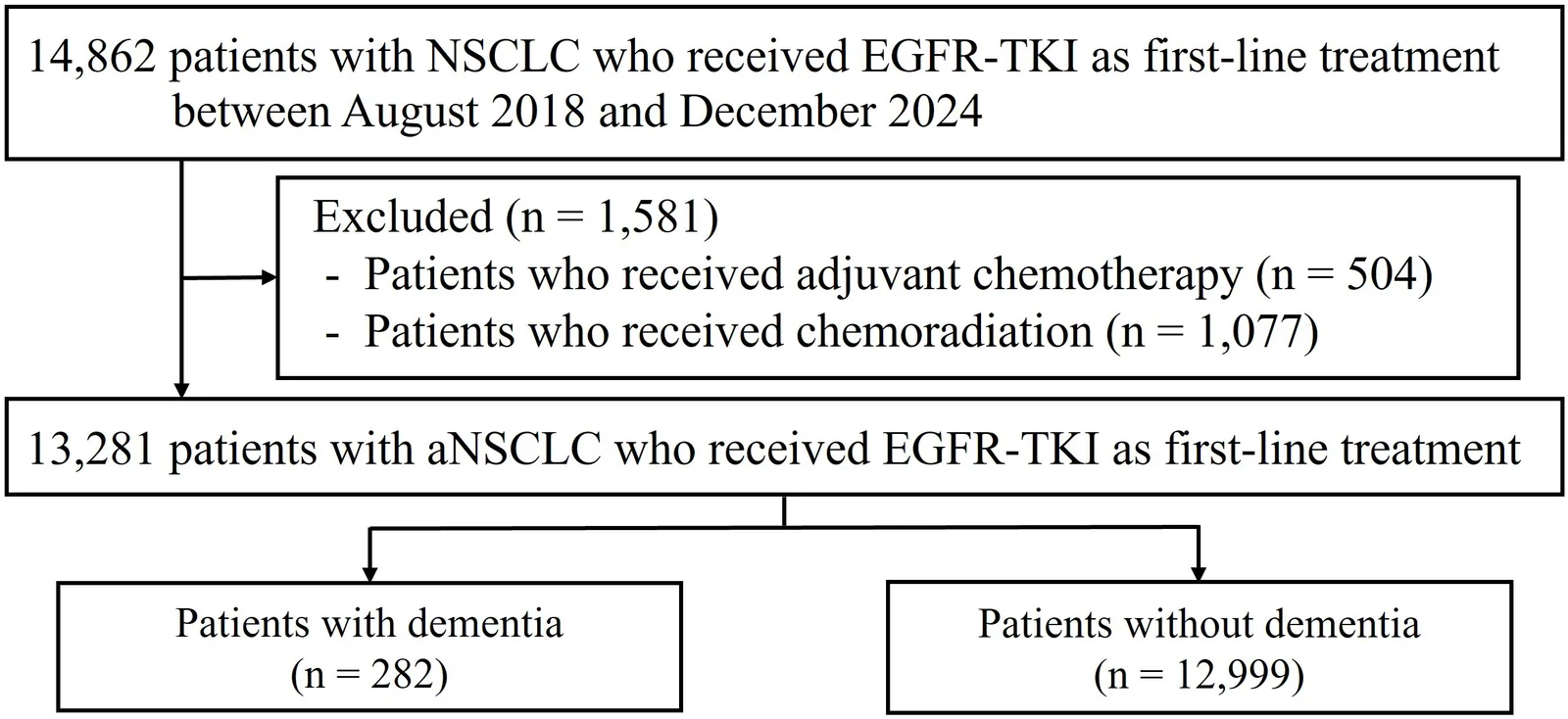
Patient selection flowchart for patients with advanced NSCLC who received first-line EGFR-TKI therapy. EGFR-TKI: epidermal growth factor receptor tyrosine kinase inhibitor; NSCLC: non–small cell lung cancer.

Baseline demographic and clinical characteristics of the study population according to coexisting dementia status are summarized in Table 1. Compared with patients without dementia, those with dementia were older (median age: 81 vs. 74 years) and more likely to be female (72.3% vs. 64.0%). The burden of comorbidities, as assessed using the CCI, differed between the groups, with a higher prevalence of more advanced comorbidities among patients with dementia. Functional impairment, reflected by a BI <100, was more common in patients with dementia than in those without dementia. Hypertension and dyslipidemia were more common among patients with dementia. Overall, patients with coexisting dementia represented a distinct clinical subgroup characterized by older age, greater functional impairment, and a higher burden of comorbidities at the initiation of EGFR-TKI therapy.

**Table 1.** Demographic and Clinical Characteristics of Patients (n = 13,281).

Patient characteristics	With dementia (n = 282)	Without dementia (n = 12,999)
Basic characteristics		
Male, n (%)	78 (27.7)	4,682 (36.0)
Female, n (%)	204 (72.3)	8,317 (64.0)
Age (years), median (IQR)	81.00 (77.00-85.00)	74.00 (68.00-80.00)
BMI (kg/m^2^)^a^, median (IQR)	20.82 (18.04-22.79)	21.71 (19.42-24.09)
Smoking status^a^, n (%)		
Current/past	57 (22.0)	3,916 (34.7)
Never	202 (78.0)	7,381 (65.3)
Charlson Comorbidity Index, n (%)		
0-2	231 (81.9)	11,485 (88.4)
≥3	51 (18.1)	1,514 (11.6)
Barthel Index^a^, n (%)		
=100	98 (53.8)	7,298 (82.8)
<100	84 (46.2)	1,520 (17.2)
Underlying conditions, n (%)		
Hypertension	152 (53.9)	4,909 (37.8)
Dyslipidemia	73 (25.9)	2,751 (21.2)
Calendar date, n (%)		
August 1, 2018-December 31, 2020	109 (38.7)	4,819 (37.1)
January 1, 2021-December 31, 2024	173 (61.3)	8,180 (62.9)

^a^BMI, smoking status, and Barthel Index data were missing for 28.4%, 13.0%, and 32.2% of the total number of patients, respectively. BMI: body mass index; IQR: interquartile range.

Overall, osimertinib was the predominant first-line EGFR-TKI, accounting for 83.3% of prescriptions (11,065/13,281), followed by afatinib (854/13,281 [6.4%]), gefitinib (790/13,281 [5.9%]), erlotinib (564/13,281 [4.2%]), and dacomitinib (8/13,281 [0.1%]). Among the patients with coexisting dementia, osimertinib was the most frequently used initial EGFR-TKI (223/282 [79.1%]), whereas only a small proportion of patients received afatinib (8/282 [2.8%]), gefitinib (39/282 [13.8%]), or erlotinib (12/282 [4.3%]).

### Association between baseline dementia and all-cause mortality

The median follow-up duration was 264 days (IQR 77-614) among patients with coexisting dementia and 470 days (IQR 175-895) among those without dementia. During follow-up, 72 deaths occurred in the dementia group and 2315 in the nondementia group. The Kaplan-Meier–estimated 3-year mortality rates were 41.9% and 30.8% in the dementia and nondementia groups, respectively (Figure 2A). Multivariable Cox proportional hazards regression analysis adjusted for baseline covariates revealed that coexisting dementia at baseline was associated with an increased risk for all-cause mortality compared with no dementia (HR 1.35, 95% CI 1.06-1.73; p = 0.016). In the complete-case sensitivity analysis, 8,549 patients were included, comprising 169 patients with coexisting dementia and 8,380 patients without dementia. Although the CI included the null value, the direction of the association between coexisting dementia and all-cause mortality was consistent with that observed in the primary analysis based on multiple imputation (HR 1.26, 95% CI 0.92-1.71; p = 0.148).

**Figure 2.**
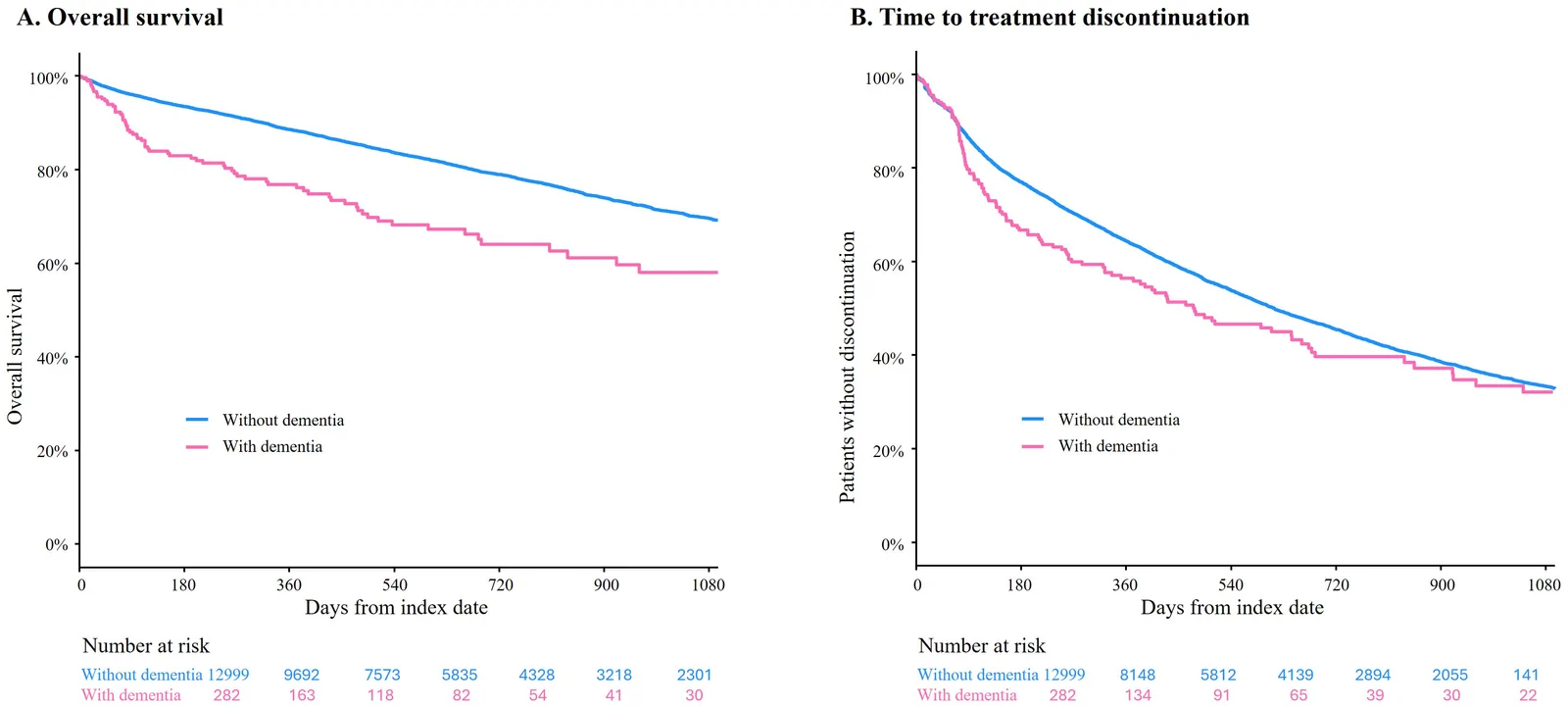
Kaplan-Meier curves for overall survival and time to treatment discontinuation according to coexisting dementia status.

Detailed results of the multivariable Cox proportional hazards analysis are reported in Table 2. The association between coexisting dementia and all-cause mortality was comparable with that observed for male sex and stronger than that of most other baseline clinical factors included in the model. While increasing age, higher comorbidity burden, and functional impairment were independently associated with mortality, coexisting dementia remained one of the strongest predictors of poor prognosis.

**Table 2.** Associations between Clinical Factors and Mortality in All Patients.

Clinical factors	HR (95% CI)	p-Value
Dementia (ref: no dementia)	1.35 (1.06-1.73)	0.016
Male (ref: female)	1.35 (1.22-1.49)	<0.001
Age - per 10-year increase	1.02 (1.01-1.02)	<0.001
Body mass index - per 1 kg/m^2^ increase	0.96 (0.95-0.97)	<0.001
Smoking status, current/former (ref: never)	1.06 (0.95-1.17)	0.325
Charlson Comorbidity Index ≥3 (ref: 0-2)	1.31 (1.16-1.48)	<0.001
Barthel Index = 100 (ref: <100)	0.55 (0.49-0.63)	<0.001
Hypertension (ref: no hypertension)	1.14 (1.04-1.25)	0.004
Dyslipidemia (ref: no dyslipidemia)	0.91 (0.82-1.01)	0.089
Calendar date ≥January 1, 2021 (ref: < January 1, 2021)	1.14 (1.05-1.23)	0.003

CI: confidence interval; HR: hazard ratio; ref: reference.

### Risk factors for all-cause mortality stratified according to baseline dementia status

Clinical risk factors associated with all-cause mortality were evaluated using multivariable Cox proportional hazard regression models, separately stratified according to baseline dementia status (Table 3).

**Table 3.** Associations between Clinical Risk Factors and Mortality by Dementia Status.

Clinical factors	With dementia		Without dementia	
	HR (95% CI)	p-Value	HR (95% CI)	p-Value
Male (ref: female)	1.59 (0.90-2.79)	0.112	1.33 (1.20-1.47)	<0.001
Age - per 10-year increase	1.00 (0.96-1.04)	0.872	1.02 (1.01-1.02)	<0.001
Body mass index - per 1 kg/m^2^ increase	0.97 (0.89-1.05)	0.466	0.96 (0.95-0.97)	<0.001
Smoking status, current/former (ref: never)	0.99 (0.52-1.87)	0.967	1.07 (0.96-1.19)	0.222
Charlson Comorbidity Index ≥3 (ref: 0-2)	0.89 (0.45-1.76)	0.738	1.33 (1.18-1.50)	<0.001
Barthel Index = 100 (ref: <100)	0.48 (0.27-0.83)	0.013	0.57 (0.51-0.64)	<0.001
Hypertension (ref: no hypertension)	0.92 (0.56-1.53)	0.759	1.14 (1.04-1.26)	0.004
Dyslipidemia (ref: no dyslipidemia)	1.04 (0.57-1.89)	0.910	0.90 (0.81-1.01)	0.067
Calendar date ≥January 1, 2021 (ref: < January 1, 2021)	0.85 (0.51-1.40)	0.518	1.14 (1.05-1.24)	0.002

CI: confidence interval; HR: hazard ratio; ref: reference.

Among patients with coexisting dementia, functional status emerged as a primary factor associated with mortality. Specifically, patients with a BI of 100 had a lower risk for death than those with an impaired functional status (HR 0.48, 95% CI 0.27-0.83; p = 0.013). In contrast, sex, age, BMI, smoking status, higher CCI score, hypertension, dyslipidemia, and the calendar date of treatment initiation were not associated with mortality. The 95% CIs for several estimates were wide, reflecting the limited sample size and the number of events among patients with dementia. In the complete-case sensitivity analysis among patients with coexisting dementia, 169 patients were included. The association between preserved functional status and lower all-cause mortality was consistent with that observed in the primary analysis based on multiple imputation (HR 0.29, 95% CI 0.15-0.55; p < 0.001).

Among patients without dementia, multiple clinical factors were independently associated with all-cause mortality. Male sex, older age, higher CCI score, and hypertension were associated with an increased risk for death, whereas higher BMI and preserved functional status (BI = 100) were associated with a reduced mortality risk. Smoking status and dyslipidemia were not associated with mortality in this subgroup.

### Assessment of medication adherence and treatment continuation

Adherence to EGFR-TKI therapy was consistently lower in patients with coexisting dementia than in those without dementia. At 1 year, the median MPR was 0.847 (IQR 0.512-0.995) in patients with dementia and 0.934 (IQR 0.609-1.000) in those without dementia. At 3 years, the corresponding median MPR values were 0.809 (IQR 0.477-0.978) and 0.849 (IQR 0.521-0.986), respectively. The proportion of patients with good adherence, defined as MPR ≥0.80, was lower among patients with dementia than among those without dementia at both 1 year (55.0% vs. 64.3%) and 3 years (51.1% vs. 54.5%).

The Kaplan-Meier–estimated treatment continuation rates at 1 year were 56.5% and 64.0% in the dementia and nondementia groups, respectively, whereas the corresponding 3-year continuation rates were 32.1% and 33.0% (Figure 2B). The Kaplan-Meier curves suggested earlier treatment discontinuation in patients with dementia, particularly during the earlier phase of follow-up. However, in the multivariable analysis, coexisting dementia was not independently associated with TTD (HR 1.16, 95% CI 0.97-1.38; p = 0.106), although the Kaplan-Meier curves and point estimate suggested a tendency toward earlier treatment discontinuation in patients with dementia.

## Discussion

In this study, coexisting dementia was associated with an increased risk of all-cause mortality among patients with EGFR-m NSCLC treated with first-line EGFR-TKIs. Other factors, including older age, higher comorbidity burden, and impaired functional status, were also independently associated with mortality. In the stratified analyses, preserved functional status (BI = 100) was associated with lower mortality among patients with dementia, whereas other baseline factors were not clearly associated with the outcomes in this subgroup. In contrast, among patients without dementia, male sex, older age, higher comorbidity burden, and hypertension were associated with increased mortality. These results suggest that patients with dementia may represent a clinically vulnerable subgroup in which functional capacity is particularly important during EGFR-TKI treatment. Consistent with this interpretation, patients with dementia also showed lower medication adherence and a tendency toward earlier treatment discontinuation during follow-up, although the association with TTD was not significant after multivariable adjustment.

Several factors may explain the observed association between dementia and mortality in this study. Dementia impairs prospective memory and executive function, which are essential for self-management of oral anticancer therapy. In this study, patients with dementia showed lower medication adherence, as reflected by lower MPR values and a lower proportion of patients with MPR ≥0.80 at both 1 year and 3 years. Patients receiving EGFR-TKIs must adhere to daily dosing schedules, recognize adverse events, and maintain regular outpatient visits. Cognitive impairment may be associated with missed doses, unplanned treatment interruptions, delayed toxicity reporting, and reduced dose intensity. Adherence to oral anticancer agents has been reported to be influenced by cognitive and psychosocial factors ^(8), (23), (24)^, and lower adherence may represent one aspect of the vulnerability observed among patients with dementia during EGFR-TKI treatment. In addition, cognitive decline may complicate shared decision-making and shift treatment goals toward less intensive approaches ^(25)^. Previous studies have reported that patients with cancer and coexisting dementia have poorer survival and are less likely to receive guideline-concordant therapies ^(6), (26)^. 

However, the interpretation of adherence and treatment-continuation findings requires caution. Although patients with coexisting dementia had lower MPR values, coexisting dementia was not significantly associated with TTD after multivariable adjustment. This apparent discrepancy may reflect differences in the aspects of treatment behavior captured by these measures. MPR was used as a claims-based proxy for medication adherence and represents the proportion of days covered by EGFR-TKI prescriptions during the observation period, whereas TTD is a broader treatment-continuation endpoint based on subsequent therapy, a prolonged treatment gap, or death. Thus, lower adherence may be observed even in the absence of a clearly shorter TTD as defined in this study. Therefore, the higher mortality observed among patients with dementia should not be interpreted as being explained solely by earlier treatment discontinuation. Rather, lower adherence may represent one aspect of the vulnerability associated with poorer survival in this population, alongside impaired functional status, frailty, comorbidity burden, unmeasured cancer severity, caregiver support, and difficulties in recognizing or managing adverse events.

Importantly, this study showed that preserved functional status was associated with lower mortality among patients with dementia. This finding suggests that prognosis in this population may be influenced not only by pharmacologic treatment but also by the maintenance of physical function. Patients with dementia are particularly vulnerable to progressive functional decline due to cognitive impairment, apathy, reduced initiative, and decreased engagement in activities of daily living. Such changes may contribute to physical deconditioning, sarcopenia, and frailty, which may in turn affect resilience during cancer treatment. These considerations highlight the clinical importance of assessing and monitoring activities of daily living in this population. Interventions aimed at maintaining functional capacity, including rehabilitation, physical activity support, and nutritional management, may be relevant components of supportive care for these patients. Multidisciplinary collaboration among physicians, nurses, rehabilitation specialists, pharmacists, and social workers is likely to be important to address modifiable factors such as mobility limitations, frailty, nutritional decline, and caregiver burden. While appropriate medication management remains important—particularly for oral targeted therapies such as EGFR-TKIs—maintaining functional capacity appears to be an important component of care in this vulnerable population.

This study has practical implications for oncology pharmacy practice. First, a brief cognitive assessment at the initiation of EGFR-TKIs may help identify patients who could require closer treatment support. Periodic reassessment during treatment may also be useful, particularly for older adults. Second, structured adherence support should be incorporated into care plans for patients with cognitive impairment. Pharmacist-led interventions may include simplified dosing instructions, one-dose-per-package dispensing, pill organizers, automated dispensing systems, written and visual medication schedules, or clearly defined toxicity action plans. Proactive follow-up by telephone or digital tools could be considered as a potential strategy to support adherence and early detection of adverse events. Finally, a comprehensive medication review may be useful in routine care. Although these strategies were not directly evaluated in this study, they may represent practical considerations for supporting the safe and sustained use of oral targeted therapy in patients with cognitive impairment. Patients with dementia frequently receive multiple concomitant medications, increasing the risk of polypharmacy and clinically significant drug–drug interactions ^(27), (28), (29)^. Careful medication reconciliation at the initiation of EGFR-TKI therapy may help to identify potentially inappropriate medications ^(30)^. When appropriate, deprescribing unnecessary agents may help reduce treatment complexity and support continued EGFR-TKI use ^(31), (32)^. Simplifying medication regimens may also reduce caregiver burden.

This study had some limitations. First, because of its observational design and use of administrative claims data, residual confounding may remain. In particular, patients with coexisting dementia represented only 2.1% of the study population, and information on dementia severity was not available in the database. Moreover, detailed oncological variables, including pathological stage, metastatic burden, performance status, and EGFR mutation subtype, could not be assessed. Therefore, unmeasured differences in dementia severity and cancer-related characteristics may have influenced the observed association, and the findings should be interpreted cautiously. Second, coexisting dementia was identified using ICD-10 diagnosis codes. Such claims-based definitions may have led to misclassification, particularly underascertainment of mild or undiagnosed cases. If this misclassification were nondifferential, it would be expected to bias the association toward the null. Third, adherence and treatment continuation were assessed using claims-based proxy measures rather than direct measures of medication-taking behavior and therefore may not fully capture the complexity of treatment management in routine practice. Finally, the number of patients with dementia was relatively small, and the 95% CIs in the subgroup analyses were wide, reflecting limited statistical precision. Therefore, these subgroup-specific estimates should be interpreted cautiously, as the study may have been underpowered to detect modest differences.

In conclusion, coexisting dementia was associated with poorer survival among patients with EGFR-m NSCLC treated with EGFR-TKIs. Functional status appeared to be an important prognostic factor in patients with dementia. These findings suggest that systematic cognitive assessment and pharmacist-led multidisciplinary support may be important for managing targeted oral therapy in patients with cognitive impairment.

## Article Information

### Acknowledgments

We would like to thank Editage (www.editage.jp) for English language editing.

### Author Contributions

Masaki Sogame and Yasutaka Ihara contributed equally to this work. Conceptualization: Masaki Sogame, Yasutaka Ihara, Shoichiro Yamamoto, Maya Shimasaki, and Shinji Akiyama. Data curation: Yasutaka Ihara. Formal analysis: Yasutaka Ihara. Funding acquisition: Yasutaka Ihara and Shinji Akiyama. Investigation: Masaki Sogame, Yasutaka Ihara, Shoichiro Yamamoto, and Maya Shimasaki. Methodology: Yasutaka Ihara. Project administration: Yasutaka Ihara. Resources: Shinji Akiyama. Software: Yasutaka Ihara. Supervision: Shinji Akiyama. Validation: Masaki Sogame, Shoichiro Yamamoto, and Maya Shimasaki. Visualization: Yasutaka Ihara. Writing – original draft: Yasutaka Ihara and Masaki Sogame. Writing – review and editing: Yasutaka Ihara, Masaki Sogame, Shoichiro Yamamoto, Maya Shimasaki, Tomohisa Kurata, and Shinji Akiyama.

### Conflicts of Interest

Yasutaka Ihara was affiliated with Daiichi Sankyo until December 2024. Shoichiro Yamamoto has received lecture fees from Merck Sharp & Dohme over the past 36 months. The other authors have no conflicts of interest to declare.

### Ethical Considerations

This study was performed in accordance with the Declaration of Helsinki. Ethics approval was waived for this human subjects study because the Japanese Ethical Guidelines for Medical and Biological Research Involving Human Subjects are not applied to clinical studies that use data anonymized before the start of the study. The data used in this study were anonymized before the start of the study by Medical Data Vision Co., Ltd. Adult participant consent was not required because all records were fully deidentified at the source and could not be linked back to individual patients; written informed consent was likewise not required.

### Dataset Availability

The dataset analyzed in this study was purchased under a data use license from Medical Data Vision Co., Ltd. (MDV). Because the data are proprietary to MDV, they are not publicly available. Access to the raw, patient-level data requires a separate paid agreement with MDV. Researchers who meet the criteria for access to confidential data may contact MDV (https://www.mdv.co.jp/) or the corresponding author for information on data access procedures. Aggregated results and analysis code are available from the corresponding author upon reasonable request and with permission from MDV.
